# The link between stress disorders and autonomic dysfunction in muscular dystrophy

**DOI:** 10.3389/fphys.2014.00025

**Published:** 2014-01-29

**Authors:** Rasna Sabharwal

**Affiliations:** Department of Internal Medicine, University of Iowa Carver College of MedicineIowa City, IA, USA

**Keywords:** heart rate variability, sympathetic nervous system, parasympathetic nervous system, baroreceptor reflex, neuromuscular disease, depression, anxiety

## Abstract

Muscular dystrophy is a progressive disease of muscle weakness, muscle atrophy and cardiac dysfunction. Patients afflicted with muscular dystrophy exhibit autonomic dysfunction along with cognitive impairment, severe depression, sadness, and anxiety. Although the psychological aspects of cardiovascular disorders and stress disorders are well known, the physiological mechanism underlying this relationship is not well understood, particularly in muscular dystrophy. Therefore, the goal of this perspective is to highlight the importance of autonomic dysfunction and psychological stress disorders in the pathogenesis of muscular dystrophy. This article will for the first time—(i) outline autonomic mechanisms that are common to both psychological stress and cardiovascular disorders in muscular dystrophy; (ii) propose therapies that would improve behavioral and autonomic functions in muscular dystrophy.

Muscular dystrophy is a devastating neuromuscular disorder that is currently without an effective therapy. It is characterized by progressive increase in muscle weakness and muscle wasting. Myocardial disease, manifesting predominantly as dilated cardiomyopathy (DCM) and congestive heart failure or conduction system abnormalities have been observed in many muscular dystrophies. Evidence of an association between vulnerability to lethal arrhythmias and signs of either increased sympathetic or reduced parasympathetic tone as quantitative markers of autonomic activity in muscular dystrophy are becoming increasingly recognized in patients and animals (Groh, 2012; Russo et al., 2012).

The physical and social hardships associated with the disease often lead to psychological stress disorders such as depression and anxiety, in muscular dystrophy patients. Psychological stress affects both mental and physical health. Depressed patients with or without a history of cardiovascular pathology display signs such as elevated heart rate (HR), reduced HR variability (HRV), and increased physiological reactivity to environmental stressors which may cause predisposition to cardiovascular events. Converging evidence from experimental and epidemiological studies indicate that there is a bidirectional association between psychological stress and cardiac risk, such that presence of one increases the likelihood of developing the other (Penninx et al., 2001; Freedland et al., 2003; Johnson and Grippo, 2006). Although psychological stress disorders are prevalent in patients with muscular dystrophy, their physiological and pathophysiological consequences on disease progression have received little attention. The focus of this perspective is to demonstrate that synergistic actions of autonomic dysregulation and psychological stress may exacerbate disease progression in muscular dystrophy and identify strategies to yield better therapeutic outcomes.

## Autonomic dysregulation in muscular dystrophy

Evidence of aberrant autonomic signaling has been demonstrated in both patients and animals with Duchenne, Becker, myotonic type 1 (DM1) muscular dystrophy. HRV, as a quantitative marker of parasympathetic tone, is decreased in muscular dystrophies (Politano et al., 2008; Inoue et al., 2009; Della Marca et al., 2010). It has been suggested that activation of the renin angiotensin system (RAS) contributes to the pathological changes occurring in muscular dystrophy (Cohn et al., 2007; Sun et al., 2009; Sabharwal et al., 2010a; Cabello-Verrugio et al., 2012). Previous work has provided evidence of autonomic dysregulation and loss of exercise-induced sympatholysis in muscular dystrophy (Sander et al., 2000). Furthermore, treatment of muscular dystrophy patients with angiotensin converting enzyme inhibitors (ACEi) and β-blockers has been shown to delay onset of DCM (Dubuc et al., 2005; Blain et al., 2013). These studies led us to determine the importance of autonomic dysregulation in pathogenesis of muscular dystrophy. We did so by utilizing an established mouse model of limb girdle muscular dystrophy (LGMD).

Dystrophin glycoprotein complex (DGC) is composed of multiple proteins that form a physical linkage between the intracellular and extracellular matrix. Sarcoglycans are important component of the DGC that protects cell membrane against forces generated during muscle contraction (Straub et al., 1998). Mutation in sarcoglycan delta (Sgcd) causes LGMD-2F. We found that young Sgcd deficient mice (Sgcd^−/−^) exhibit severe autonomic dysregulation (reduced HRV, impaired baroreflex function, sympatho-vagal imbalance) before left ventricular (LV) dysfunction (Sabharwal et al., 2010b). Furthermore, we found that the severity of autonomic dysregulation in young Sgcd^−/−^ mice predicts severity of LV dysfunction and mortality at older ages (Sabharwal et al., 2010b). We speculate that the combined effects of chronic autonomic dysfunction and psychological stress provoke arrhythmias in muscular dystrophy; the latter has been recognized as a common cause of mortality in muscular dystrophy (Groh, 2012; Russo et al., 2012).

## Psychological stress in muscular dystrophy

The association of intellectual impairment with Duchenne muscular dystrophy has long been recognized. Recent studies have demonstrated psychiatric disorders such as autism, dysthymic and clinical depression are fairly common in patients afflicted with DM1, Duchenne, LGMD, and facioscapulohumeral muscular dystrophies (Fitzpatrick et al., 1986; Douniol et al., 2009; Winblad et al., 2013). Children with muscular dystrophy not only face inevitable deterioration of physical functioning, but also become susceptible to emotional and behavioral problems, mental alteration mainly characterized by poor verbal Intelligence Quotient, memory, and cognitive deficits, and reading disabilities (Billard et al., 1992; Polakoff et al., 1998). These deficits are without histopathological abnormalities in the brain (Dubowitz and Crome, 1969; Billard et al., 1992). The *mdx* mouse is a widely used experimental model of human Duchenne muscular dystrophy. In addition to skeletal muscle weakness and reduced locomotor activity, *mdx* mice exhibit disruption in synaptic plasticity, reduced long-term potentiation in Purkinje cells, learning, and memory deficits along with enhanced anxiety-related and defensive freezing behavior (Anderson et al., 2004; Sekiguchi et al., 2009).

In DM1, heart conduction defects, endocrine dysfunction and brain abnormalities such as cerebral atrophy and white matter lesions are fairly common (Meola and Sansone, 2007). DM1 is associated with cognitive deficits, depression, anxiety, mood, and personality disorders (Meola and Sansone, 2007). Similarly, mice with deletion of muscleblind-like 1 (Mbnl1), a RNA splice regulator that causes DM1, exhibit cognitive impairment, learning, and memory deficits, and behavioral abnormalities (depression, autism, anxiety) (Matynia et al., 2012).

Peripartum cardiomyopathy is LV dysfunction that presents toward the end of pregnancy or in the months just after delivery. Women afflicted with muscular dystrophy and psychological stress disorders are at a greater risk to develop peripartum cardiomyopathy. Recently, two cases of postpartum cardiomyopathy have been reported in previously asymptomatic carriers of Duchenne muscular dystrophy (Davies et al., 2001; Cheng and Prior, 2013). Women with peripartum cardiomyopathy have reduced levels of signal transducer and activator of transcription 3 (STAT3), increased cathepsin D, and decreased expression of manganese superoxide dismutase; the combination of which causes apoptosis, impaired angiogenesis, and oxidative stress. Bromocriptine, that blocks the release of prolactin from the pituitary gland, has been reported to decrease morbidity and mortality in this condition (Sliwa et al., 2010), although this is yet to be confirmed.

Whilst physiological mechanisms of psychological stress are largely unknown, these association studies demonstrate high prevalence of stress disorders in muscular dystrophies. There is mounting evidence that psychological stress plays a critical role in triggering cardiac arrhythmias and sudden cardiac death. Therefore, it becomes imperative to determine the underlying mechanisms of these dysfunctions.

## Proposed mechanisms of autonomic dysfunction and psychological stress in muscular dystrophy

Depression has been characterized by activation of the sympathetic nervous system and withdrawal of parasympathetic tone to the heart, increased resting HR and reduced HRV (Lahmeyer and Bellur, 1987; Barton et al., 2007). In humans, 24-h electrocardiogram provides a useful strategy for investigating autonomic consequences of depression and predictors of mortality (Aronow et al., 1996; Guzzetti et al., 2005). Cardiac norepinephrine spillover, activation of sympathetic nervous system and reduced neuronal reuptake of norepinephrine predispose to the development of cardiac arrhythmias in psychological disorders (Esler et al., 2004; Barton et al., 2007). Chronic stress can also evoke arrhythmias by altering stability of cardiac repolarization (Carney et al., 2003; Lampert et al., 2005). Lambert et al. suggest that the sympathetic neurons fire more often in multiple spike pattern in patients with panic disorders (Lambert et al., 2006).

**Figure 1:**
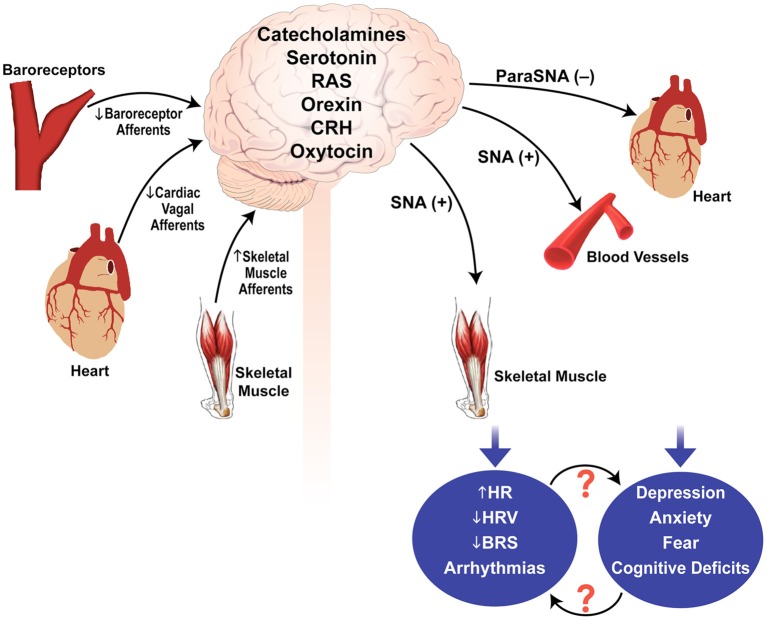
**Possible mechanisms involved in autonomic dysfunction and psychological stress in muscular dystrophy**. Sensory input by the baroreceptor afferents, cardiac vagal afferents, and skeletal muscle afferents are integrated within the brain to modulate parasympathetic and sympathetic outflows. Disturbances in the sensory inputs and/or central integration and neurotransmission may contribute to autonomic dysregulation and psychological stress in muscular dystrophy. SNA, sympathetic nerve activity; ParaSNA, parasympathetic nerve activity; RAS, renin angiotensin system; CRH, corticotrophin releasing hormone; HRV, heart rate variability; HR, heart rate; BRS, baroreflex sensitivity; (−), inhibition; (+), excitation.

Some of the reflexes that could be important in regulating sympathetic and parasympathetic outflow in muscular dystrophy are baroreceptor reflexes, skeletal muscle afferent reflex, and cardiac vagal afferent reflex (Figure 1). Baroreceptors are mechanosensitive nerve endings located in carotid sinuses and aortic arch that function as blood pressure sensors. Changes in baroreceptor activity evoke reflex changes in parasympathetic and sympathetic activity (Chapleau et al., 2001; Head and Mayorov, 2001; Stauss, 2002). Reduced baroreflex gain can contribute to cardiovascular morbidity and mortality *via* reduction in parasympathetic activity, an increase in sympathetic activity, or both. There are several reports whereby reduced baroreflex gain and depression increase the risk of ventricular fibrillation (Billman et al., 1982; Schwartz et al., 1988; Watkins and Grossman, 1999). Rats exposed to a series of chronic mild stressors exhibit anhedonia (an essential feature of human depression), reduced baroreflex function, elevated HR, decreased HRV, and exaggerated pressor and HR responses to air jet stress (Grippo et al., 2002, 2008). Mechanoreceptors located in the heart and cardiopulmonary region sense changes in central blood volume through their sensitivity to cardiac and vascular distension. In heart failure or DCM (as seen in advanced stages of muscular dystrophy), the mechanosensitivity of cardiac vagal afferents is severely depressed which can contribute to an increase in sympathetic tone, decrease in parasympathetic tone, and fluid retention (Walgenbach and Shepherd, 1984; DiBona and Sawin, 1995). Another neural mechanism that is likely to be important in muscular dystrophy is the somatic afferent reflex. Group III and IV muscle afferents are activated by changes in pH, inflammatory mediators, oxidative stress and/or by abnormal mechanical coupling between muscle and sensory nerve endings to evoke reflex increases in sympathetic activity, inhibition of parasympathetic activity, and decrease in baroreflex gain (Delliaux et al., 2009; Kaufman, 2012).

Stressful stimuli of different modalities activate sympatho-adrenal and hypothalamic-pituitary (HPA) axes (Badoer, 2001). It is now well established that the dorsomedial hypothalamus is a key area involved in integrating neurally-mediated cardiovascular responses to psychological stress. This area receives synaptic inputs from many regions of the brain that are activated during stress including periaqueductal gray, amygdala, raphe pallidus, rostroventral, and caudal portions of the lateral medulla, hippocampus, and the nucleus of the solitary tract (Cechetto and Chen, 1990; Hunt et al., 2010; Xavier et al., 2013). Some of the neurotransmitters involved in cardiovascular and behavioral responses to stress are serotonin, catecholamines, dopamine, histamine, oxytocin, vasopressin, angiotensin II, corticotrophin releasing hormone, the cytokines IL-1β, and TNF-α etc. (Mayorov and Head, 2003; Wsol et al., 2008). It is noteworthy that several proteins belonging to the DGC and involved in muscular dystrophies (dystroglycans, dystrophin, sarcoglycans) have now been identified in forebrain, midbrain, and brainstem (Anastasi et al., 2012; Waite et al., 2012).

It is important to emphasize that mechanisms of autonomic dysfunction or psychological stress in muscular dystrophy are yet to be identified. Figure 1 thus provides a simplistic overview of possible pathways/mechanisms that may contribute to the pathogenesis of this disease.

## Therapeutic targets to improve autonomic function and reduce psychological stress

The RAS is activated in muscular dystrophy (Sun et al., 2009; Sabharwal et al., 2010a; Cabello-Verrugio et al., 2012). RAS is comprised of two axes—vasoconstrictor angiotensin II (Ang II) acts primarily via Ang II type 1 receptors (AT_1_R) and the vasodilator Ang-(1–7) peptide acting on Mas receptors (Bader et al., 2012). Ang II also acts on type 2 receptors (AT_2_R). Angiotensin receptor blockers (ARBs) and ACEi are used clinically in muscular dystrophy and dilated cardiomyopathy (Dubuc et al., 2005; Cohn et al., 2007). It is well known that Ang II is a major stress hormone (DuboYang et al., 1993). ARBs and ACEi are also used in treating conditions like mood disorders, depression, and neurodegenerative and traumatic disorders of the brain (Bostwick, 2010). Systemic administration of candesartan/losartan can abrogate behavioral responses to acute and long-term isolation stress (Baiardi et al., 2004; Armando et al., 2007), cold restraint stress (Bregonzio et al., 2008), and forced swim test (Martin et al., 1990) in rodents. ARBs and ACEi have also been shown to be effective in reducing brain inflammation associated with depression (Young et al., 2003; Munoz et al., 2006). Psychological stress reduces expression of central AT_2_R receptors and animals lacking AT_2_R develop anxious behavior (Hein et al., 1995; Ichiki et al., 1995). These studies suggest that centrally acting AT_2_R agonists may serve as potential anxiolytic agents. More recently, Ang-(1–7) has also been suggested to exert anxiolytic actions. Bild et al demonstrated that rats treated with Ang-(1–7) performed better in the maze test than the untreated animals (Bild and Ciobica, 2013). Furthermore, mice lacking Mas receptors display increased anxiety (Walther et al., 1998), and activation of the Ang-(1–7)/Mas axis attenuates stress-induced tachycardia in rats (Martins Lima et al., 2013). These studies suggest that drugs targeting the RAS [Ang-(1–7)/Mas, AT_2_R agonists, ACEi, or ARBs] may improve outcomes in muscular dystrophy not only through their direct autonomic/cardiovascular effects but also via their anxiolytic actions.

Serotonin (5-hydroxytryptamine, 5-HT) is a neurotransmitter that modulates stress responses by interacting with the HPA axis and sympathetic nervous system (Chrousos, 2009). Selective serotonin reuptake inhibitors (SSRI) and serotonin norepinephrine reuptake inhibitors (SNRI) are used for treatment of depression and anxiety (Glassman et al., 2002; Trivedi et al., 2006). SSRIs exert their effects via modulating monoaminergic signaling or via their anti-inflammatory actions (Walker, 2013). SSRIs are considered safe even when administered to patients with serious cardiovascular diseases (Glassman et al., 2002; Taylor et al., 2005). SSRIs reduce depressive symptoms, increase HRV, reduce inflammatory markers, normalize urinary cortical excretion, and reduce plasma catecholamine levels (Glassman et al., 2002). Loss of central 5-HT neurons may cause hypersomnia in patients with myotonic dystrophy (Ono et al., 1998). SSRI therapy can also revert abnormal electromyogram in myotonic dystrophy type 1 (Chisari et al., 2009), and improve psychiatric comorbidities in Becker muscular dystrophy (Chaichana et al., 2007). Thus, SSRI therapy may improve outcomes in muscular dystrophy via multiple mechanisms.

## Summary

Muscular dystrophy is a catastrophic disease which is characterized by progressive muscle weakness and wasting, cardiomyopathy, and early mortality. Patients with muscular dystrophy are commonly afflicted with psychological disorders like depression, anxiety, cognitive deficits etc., which likely exacerbates disease progression and worsens the quality of life. Both muscular dystrophy and behavioral disorders are associated with autonomic dysregulation. In view of the devastating outcome of muscular dystrophy most attention has been directed toward improving muscle function and structure. However, treating both autonomic dysregulation and stress disorders is recommended for patients with muscular dystrophy. Additive and synergistic actions are likely to result in better therapeutic outcomes.

### Conflict of interest statement

The author declares that the research was conducted in the absence of any commercial or financial relationships that could be construed as a potential conflict of interest.
